# Clinical evaluation of a *Clematis chinensis* Osbeck–containing mouthwash for the prevention of dental caries: a randomized, controlled clinical trial

**DOI:** 10.1186/s13020-025-01258-z

**Published:** 2025-11-24

**Authors:** Yu-Rin Kim, Seoul-Hee Nam

**Affiliations:** 1 Department of Dental Hygiene, Silla University, 140 Baegyang-daero 700beon-gil, Sasang-gu, Busan, 46958 Republic of Korea; 2 Department of Dental Hygiene, College of Health Sciences, Kangwon National University, 346 Hwangjo-Gil, Dogye-Up, Samcheok-Si, Gangwon-Do 25945 Republic of Korea

**Keywords:** *Clematis chinensis* Osbeck extract, Antibacterial mouthwash, Dental caries prevention, Randomized controlled trial, *Streptococcus mutans*, Buffering capacity

## Abstract

**Background:**

Chlorhexidine is widely used as a chemical antibacterial mouthwash, but its potential side effects have spurred interest in safer natural alternatives.

**Purpose:**

This study aimed to evaluate the anticariogenic efficacy and inhibitory effects of a functional mouthwash containing *Clematis chinensis* Osbeck (*C. chinensis* Osbeck) extract in a randomized controlled clinical trial.

**Study design and methods:**

In this randomized, double-blind, controlled clinical trial conducted at Busan M Dental Clinic, 69 participants were randomly assigned to three groups: saline gargle (n = 23), chlorhexidine gargle (n = 23), and *C. chinensis* Osbeck extract gargle (n = 23). Participants used 15 mL of the assigned mouthwash four times daily for 2 weeks. The oral environment was standardized with professional scaling and a 1-week recovery period before the intervention. Clinical outcomes were evaluated using the O’Leary index and the Cariview™ test kit (AIOBIO Co. Ltd., Seoul, South Korea) at baseline, 1 week, and 2 weeks, respectively, to evaluate dental plaque acidogenicity and user satisfaction. Saliva tests (including cariogenic bacterial counts, acidogenicity, and buffering capacity) were performed using the SillHa Oral Wellness System (ARKRAY Inc., Kyoto, Japan). Subgingival plaque samples were analyzed by quantitative real-time polymerase chain reaction (PCR) analysis to detect cariogenic bacteria (*Streptococcus mutans* [*S. mutans*] and Gram-positive cariogenic bacteria [*GS* group], comprising *S. mitis*, *S. sobrinus*, and *Lactobacillus casei*). Statistical analyses were performed using analysis of variance (ANOVA) and Duncan’s post hoc test, with significance set at *P* < 0.05.

**Results:**

The *C. chinensis* Osbeck extract gargle group showed reductions in the O’Leary index score and cariogenic activity, alongside a progressive increase in user satisfaction. Saliva analysis revealed significant decreases in caries-causing bacterial numbers and acid production and improved buffering capacity, enhancing salivary defense. Only the *C. chinensis* Osbeck extract gargle group showed a significant reduction in *S. mutans* and the *GS* group. Compared with the saline and chlorhexidine groups, this group demonstrated a continuous decrease in caries risk over the 2 weeks.

**Conclusion:**

*C. chinensis* Osbeck extract significantly improved clinical parameters related to dental caries, suggesting its potential as a safe and effective natural alternative to chemical antibacterial agents for caries prevention and oral health maintenance.

**Trials registration:**

ClinicalTrials.gov, KCT0008539. Registered on June 21, 2023, https://cris.nih.go.kr/cris/search/detailSearch.do/23816).

**Supplementary Information:**

The online version contains supplementary material available at 10.1186/s13020-025-01258-z.

## Introduction

The oral cavity is vital in sustaining life by facilitating nutrient intake, initiating chewing and digestion, and contributing to speech and aesthetics [1]. Maintaining this condition is essential even in individuals with good oral health, as neglect can lead to oral diseases [2]. Furthermore, oral hygiene has been linked to systemic conditions such as cardiovascular disease, rheumatoid arthritis, and osteoporosis [3].

Dental caries is among the most common oral diseases across all age groups. Its primary causative agent, *Streptococcus mutans* (*S. mutans*), secretes glycosyltransferases (GTFs), which synthesize glucan, a polysaccharide, from sugars. This polysaccharide promotes bacterial adhesion to tooth surfaces, forming biofilms. The acid produced during this process induces enamel demineralization, leading to dental caries [4, 5]. Effective prevention involves inhibiting *S. mutans* growth, reducing GTF secretion, and preventing glucan synthesis and bacterial adherence [6]. Controlling these pathogenic mechanisms weakens biofilm structure and suppresses caries development [7].

An ideal oral antibacterial agent should selectively target pathogens while preserving normal flora. Mouthwashes are widely used for this purpose due to their ease of application and effectiveness in inhibiting bacterial growth and biofilm formation [7]. Chemical agents such as chlorhexidine, cetylpyridinium chloride (CPC), and triclosan have demonstrated vigorous antibacterial activity against primary caries-causing bacteria such as *S. mutans* [8]. However, many mouthwashes currently used to prevent and treat oral diseases contain chemical agents that may harm the human body, causing side effects such as oral mucosal irritation, tooth and soft tissue discoloration, and altered taste sensation [9]. In particular, chlorhexidine is effective for short-term use but poses a risk of opportunistic infections due to suppressing the normal oral flora during prolonged use [10]. These physiological risks and frequent adverse effects with long-term use have raised ongoing concerns regarding the safety of chemical antibacterial agents [9].

Such limitations emphasize the need for natural antibacterial agents that selectively target oral pathogens while remaining biocompatible. In response, recent studies have focused on identifying natural extracts that could minimize the side effects of chemical agents and serve as safe alternatives for preventing oral diseases [11]. For instance, *Sambucus williamsii* var. *coreana* Nakai (*S. Williamsii*) extract has demonstrated antibacterial activity by inhibiting the growth of *S. mutans*, indicating its potential as a natural anticariogenic agent [12]. Similarly, *Diospyros malabarica* stem extract has been reported to prevent biofilm formation by inhibiting *S. mutans* adhesion to the tooth surface during the biofilm formation stage [13]. Natural extracts exhibit high biocompatibility and selectively inhibit pathogenic oral bacteria, making them promising candidates for functional mouthwash formulations to prevent dental caries.

*Clematis chinensis* Osbeck (*C. chinensis* Osbeck), a perennial vine root of the genus *Clematis* (family Ranunculaceae), has traditionally been used to treat edema, neuralgia, myalgia, and migraines. Its anti-inflammatory, antibacterial, and analgesic effects have also been reported [14–16]. This medicinal plant has a long history of use in managing various conditions, including parotitis, liver disease, conjunctivitis, arthritis, and tonsillitis [17]. Given its physiological activities, *C. chinensis* Osbeck shows potential for application in oral bacterial diseases. However, studies exploring its clinical efficacy and role in improving the oral environment through antibacterial and anti-inflammatory actions remain limited.

Therefore, a systematic clinical study is warranted to assess the potential of *C. chinensis* Osbeck extract as a natural ingredient in oral hygiene products for preventing oral diseases. This study aimed to evaluate the clinical efficacy of a mouthwash containing *C. chinensis* Osbeck extract in preventing and improving dental caries by assessing its antibacterial effects against cariogenic bacteria, inhibition of biofilm acid production, and saliva parameters.

## Methods and materials

### Extraction of* C. chinensis* Osbeck

*C. chinensis* Osbeck was purchased from Cheongmyeong Co., Ltd. (Chungju, South Korea). The dried material was pulverized and extracted with 70% ethanol at 60 °C for 12 h. The extract was filtered through qualitative filter paper (Advantec No. 2, Toyo Roshi Kaisha, Ltd., Tokyo, Japan) and concentrated under reduced pressure using a rotary vacuum evaporator (N-1300E.V.S., EYELA Co., Ltd., Tokyo Rikakikai Co., Ltd., Tokyo, Japan). The concentrated extract was lyophilized using a freeze dryer (Ilshin Lab Co., Yangju-si, South Korea) at − 80 °C to obtain a powder. The final product was stored at − 20 °C and reconstituted in distilled water to prepare a mouthwash at 10 mg/mL concentration.

### Ethical consideration

This study complied with the International Council for Harmonisation of Technical Requirements for Pharmaceuticals for Human Use (ICH) guidelines. Ethical approval was obtained from the Institutional Review Board of Kangwon National University (KWNUIRB-2023-01-003-001, Chuncheon, South Korea). The trial was prospectively registered with the World Health Organization International Clinical Trials Registry Platform (WHO ICTRP) under registration number KCT0008539 (registered on June 21, 2023; https://cris.nih.go.kr/cris/search/detailSearch.do/23816). All participants were thoroughly informed about the study’s objectives, procedures, and potential risks before enrollment. Participation was voluntary, and participants could withdraw at any time without penalty. Written informed consent was obtained from all participants.

### Study participants

**Fig. 1:**
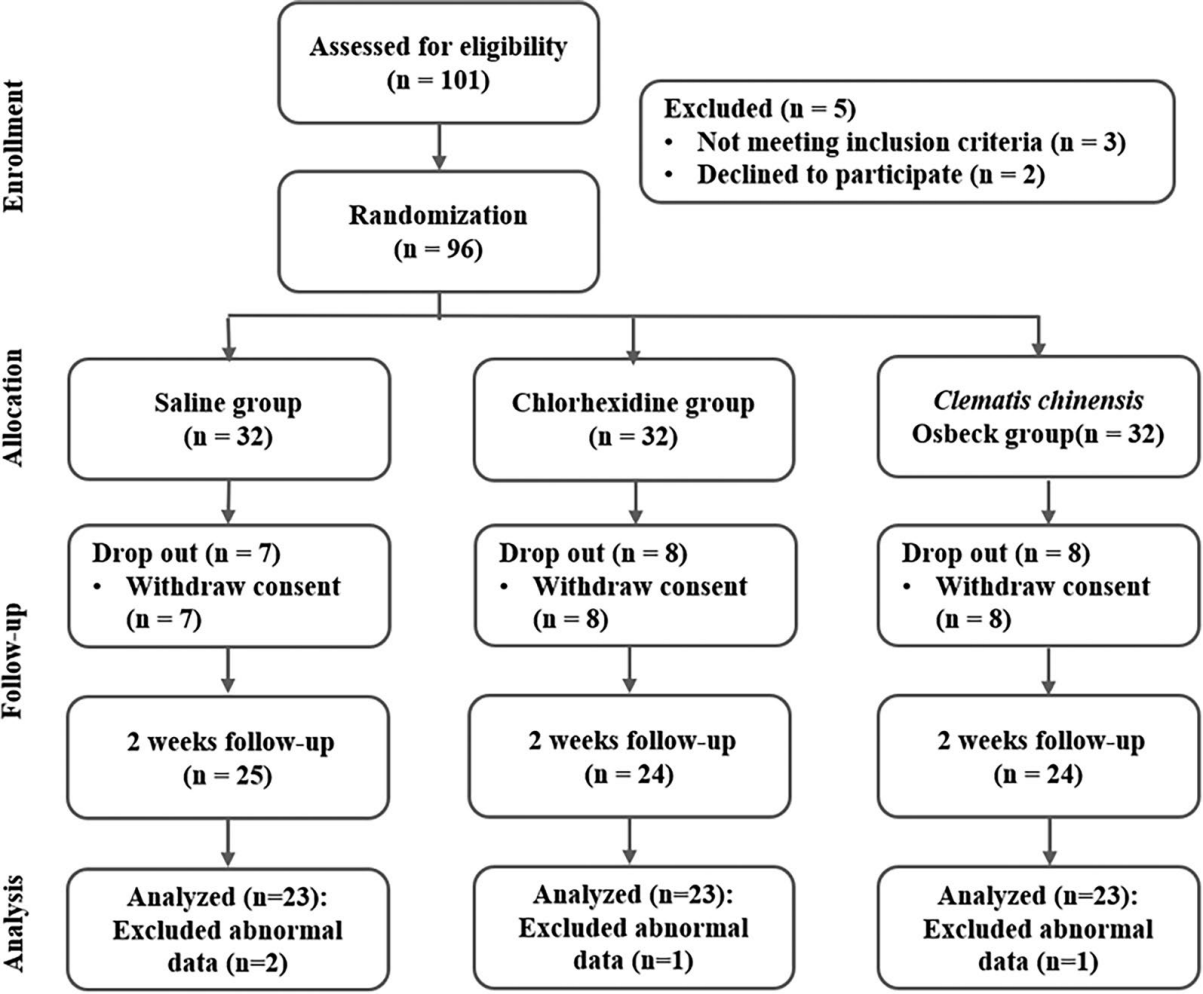
Study flow diagram

The sample size was calculated using G*Power version 3.1 software. For a repeated measures analysis of variance (ANOVA) with a within-between interaction, assuming a two-tailed significance level of 0.05, statistical power of 0.95, an effect size of 0.25, and three groups, the minimum required sample size was 54 participants. A total of 101 participants were initially recruited. After excluding five individuals who declined participation or failed to meet the inclusion criteria, 96 participants were enrolled and randomly assigned to three groups: saline gargle (n = 32), chlorhexidine gargle (n = 32), and *C. chinensis* Osbeck gargle (n = 32). During the intervention, 23 participants withdrew (saline: 7; chlorhexidine: 8; C. chinensis Osbeck: 8), and 4 datasets were excluded from analysis due to abnormal data (Fig. 1).

### Study design and protocol

This randomized, double-blind, controlled clinical trial was conducted between January and April 2023 at M Dental Clinic, Busan, South Korea. Participants were recruited from patients visiting the clinic during the study period. A dental hygienist with over 10 years of clinical experience explained the study’s objectives and procedures, and only those who voluntarily provided a written informed consent form were enrolled. Inclusion criteria required participants to have at least 16 natural teeth. Exclusion criteria were periodontitis, more than one dentinal carious lesion, severe oral conditions such as xerostomia, systemic diseases associated with halitosis (such as liver disease, kidney disease, Sjögren’s syndrome, rheumatoid arthritis), smoking, sinusitis or rhinitis, antibiotic use, tongue disorders such as glossitis or tongue cancer, and recent dental scaling within the past two months. Ultimately, 69 participants meeting the eligibility criteria were included in the final analysis.

### Clinical examination

To standardize the oral environment, participants visited M Dental Clinic (Busan) 1 week before the study for an oral examination by a dentist, and two trained dental hygienists performed light scaling. Following a 1-week recovery period, baseline clinical assessments were conducted, including dental caries–related indices (O’Leary index and cariogenic activity), participant satisfaction with mouthwash use, salivary tests for bacterial count, acid production, and buffering capacity, and evaluation of two major cariogenic bacteria.

Participants were randomly assigned to three groups. Randomization was performed using a computer-generated number table by an independent researcher not involved in participant enrollment or assessment. Group allocation was concealed using sequentially numbered, opaque, sealed envelopes to minimize selection bias. The *C. chinensis* Osbeck extract gargle group used 15 mL of a 10 mg/mL extract; the chlorhexidine group used 15 mL of chlorhexidine mouthwash; and the saline group used 15 mL of saline solution. All participants gargled for 1 min, four times daily (after meals and before bedtime). To monitor adherence, participants returned used mouthwash containers and maintained a daily usage diary, which was reviewed by study staff. Group allocation was blinded for both participants and clinical evaluators, with all mouthwash containers identical in appearance to maintain blinding. Participants received the same toothbrush and toothpaste, as well as standardized oral hygiene instructions throughout the study. Baseline measurements were taken one week after scaling, with follow-up assessments at 1 and 2 weeks post-baseline. Two trained dental hygienists, blinded to group allocation, recorded all caries-related indices under dentist supervision. Inter- and intra-examiner reliability was assessed prior to the study to ensure measurement consistency. Pre-specified criteria were applied for handling missing or abnormal data, and any deviations were documented. During the 3-week study period, participants were instructed to restrict intake of foods that could influence oral acidity and caries risk (e.g., chocolate, candy, sour foods, acidic fruits, fruit juices) and to abstain from food, beverages, and water before each clinical assessment to minimize variation in oral conditions.

### Sociodemographic characteristics

A structured, self-administered pre-survey was conducted before the study to assess the participants’ demographic profiles and ensure group compatibility. Demographic variables included sex (male/female), age (years), marital status (single/married), presence of systemic disease (yes/no), frequency of oral check-ups (regular/irregular), and previous experience with tooth brushing instruction (TBI) (yes/no). Moreover, participant satisfaction with mouthwash use was assessed using a 5-point Likert scale (1: very dissatisfied to 5: very satisfied).

### Dental caries-related indices

#### O’Leary index

The O’Leary plaque control record, developed by O’Leary, Drake, and Naylor (1972) [18], was used to assess dental plaque quantitatively. Before the evaluation, a disclosing solution was applied to all tooth surfaces for plaque visualization. Each tooth's mesial, distal, buccal, and lingual surfaces were examined. A score of 1 was assigned if plaque was present and 0 if absent. The total plaque score was calculated by dividing the number of surfaces with plaque by the total surfaces examined and converting this to a percentage. Higher percentages indicated poorer oral hygiene, while lower percentages reflected better plaque control.

#### Cariogenic activity

Cariogenic activity was evaluated using the Cariview™ assay kit (AIOBIO Co. Ltd., Seoul, Korea) according to the manufacturer's instructions. This test indirectly measures the oral cavity’s acidogenic potential through a colorimetric reaction following the culture of plaque samples collected from tooth surfaces, quantifying caries risk. Plaque was obtained from the buccal surfaces of the first molars (#16 and #36) using sterile cotton swabs, immediately immersed in a specialized culture medium, and incubated at 37 °C for 48 h. After incubation, 10 drops of indicator were added to induce a color change, which was photographed using an optical image analyzer (Allinone Bio, Seoul, South Korea, https://www.aiobio.co.kr/official.php/home/info/3974). The images were uploaded to the manufacturer's analysis system, automatically calculating the Cariview score. Scores were categorized as follows: 0.0–40.0 (low risk), 41.0–70.0 (medium risk), and 71.0–100.0 (high risk). The Cariview score corresponds to acid proportion capacity, where a higher score denotes greater caries activity.

#### Dental caries risk assessment using saliva tests

Saliva tests were consistently performed by a single trained examiner using the SillHa ST-4910 system (ARKRAY Inc., Kyoto, Japan), following the manufacturer’s instructions. Participants gargled with 3 mL of mouthwash for 10 s and expectorated into a sterile container. Saliva was collected using a dropper and applied to the seven pads of a test strip, which was then placed in the device’s strip holder. Measurement began upon closing the lid, and results were available in approximately 5 min. The system automatically assessed three parameters: cariogenic bacterial load (10⁶–10⁸ CFU/mL), acid-producing capacity (pH 6.0–8.0), and buffering capacity (pH 2.8–6.0). Buffering capacity was reverse-coded so that higher parameter values reflected poorer oral health. Device reliability was confirmed by retesting 20 randomly selected samples at one-week intervals.

#### Microbiological analysis

Subgingival microbiota samples were collected using sterilized #15 paper points inserted into the gingival sulcus at four sites: two maxillary teeth (#16 and #21) and two mandibular teeth (#36 and #41) with probing depths < 4 mm. Paper points were left in place for 10 s, then transferred to sterile 1.5 mL microcentrifuge tubes and stored at − 20 °C until DNA extraction. Genomic DNA was extracted using the AccuPrep Universal RNA Extraction Kit (Bioneer Corporation, Daejeon, South Korea), per the manufacturer’s protocol. For quantitative real-time polymerase chain reaction (qPCR) analysis, species-specific oligonucleotide sets comprising forward primer, reverse primer, and probe (Supplementary Table 1) were used with OligoMix (YD Global Life Science Co., Ltd., Seongnam, South Korea). Each 20 μL reaction mixture contained 9 μL OligoMix, 10 μL of 2 × probe qPCR mix (Takara Bio Inc., Shiga, Japan), and 1 μL of DNA template. Amplification was performed on a CFX96 Touch Real-Time PCR Detection System (Bio-Rad Laboratories, Inc., Hercules, CA, USA) under the following cycling conditions: 95 °C for 30 s (activation), followed by 40 cycles of 95 °C for 10 s (denaturation), and 62 °C for 30 s (annealing). Cycle threshold (Ct) values were analyzed using the Bio-Rad CFX Manager software (Bio-Rad Laboratories, Inc.), and bacterial copy numbers were calculated by interpolating Ct values against species-specific standard curves.

### Statistical analysis

All analyses were performed using Statistical Package for the Social Sciences (SPSS) statistical software (version 29.0, IBM Corp., Armonk, NY, USA), with statistical significance set at P < 0.05. One-way ANOVA was applied to compare continuous demographic variables among the saline, chlorhexidine, and *C. chinensis* Osbeck groups, while chi-squared tests were employed for categorical variables. Clinical caries indices, salivary diagnostics, and bacterial counts were compared using one-way ANOVA, followed by Duncan’s multiple range test for post hoc comparisons. Effect sizes were reported as eta-squared (η^2^). All tables included corresponding *P*-values, with statistical significance at 5%.

## Results

### Comparison of sociodemographic characteristics

The sociodemographic data of the saline gargle, chlorhexidine gargle, and *C. chinensis* Osbeck extract groups are summarized in Table 1. Female participants predominated in all groups, with similar mean ages of 41.09 years, 40.43 years, and 40.26 years, respectively. Most participants were married, and only individuals without systemic diseases were included. Moreover, the proportion of participants undergoing regular oral check-ups or with prior TBI experience was low across all groups. There were no significant differences in the demographic variables among the three groups (*P* > 0.05).

**Table 1:** Participant characteristics in the three groups

Characteristics		N (%)		
	Saline group (n = 23)	Chlorhexidine group (n = 23)	*Clematis chinensis* Osbeck group (n = 23)	*p*-value
Sex^*^				
Male	10 (43.5)	10 (43.5)	10 (43.5)	1.000
Female	13 (56.5)	13 (56.5)	13 (56.5)	
Age (mean ± SD)	41.09 ± 3.73	40.43 ± 9.28	40.26 ± 10.04	0.937
Marital status^*^				
Single	4 (17.4)	4 (17.4)	4 (17.4)	1.000
Married	19 (82.6)	19 (82.6)	19 (82.6)	
Systemic disease^*^				
No disease	23 (100.0)	23 (100.0)	23 (100.0)	1.000
Have a disease	0 (00.0)	0 (00.0)	0 (00.0)	
Regularity of oral check-ups^*^				
Irregular	17 (73.9)	19 (82.6)	17 (73.9)	0.722
Regular	6 (26.1)	4 (17.4)	6 (26.1)	
Tooth brushing instruction (TBI) experience^*^				
Inexperienced	16 (69.6)	18 (78.3)	19 (82.6)	0.566
Experienced	7 (30.4)	5 (21.7)	4 (17.4)	

*SD* standard deviation

^￥^*p*-values are determined by ANOVA, ^*^p-values are determined by chi-square test, (p < 0.05)

### O’Leary index, cariogenic activity, and mouthwash satisfaction analysis

Table 2 and Supplementary Fig. 1 summarize the changes in clinical indices across the saline gargle, chlorhexidine gargle, and *C. chinensis* Osbeck gargle groups. At baseline, no significant differences in the O’Leary index scores were observed among the groups. However, by week 1, both the chlorhexidine and *C. chinensis* Osbeck groups showed significantly lower scores compared to the saline group, with the extract group exhibiting a more pronounced reduction (η^2^ = 0.322, *P* < 0.001). This trend continued at week 2, where the *C. chinensis* Osbeck group demonstrated greater improvement than the chlorhexidine group (η^2^ = 0.410, *P* < 0.001). Over time, the saline and chlorhexidine groups showed increases in the O’Leary index score from baseline to week 1, though only the saline group’s increase was significant (η^2^ = 0.325, *P* < 0.001). Conversely, the *C. chinensis* Osbeck group showed a progressive decrease from week 1, significantly reducing by week 2 (η^2^ = 0.142, *P* = 0.006).

**Table 2:** Differences in dental caries-related indices and satisfaction levels among the three groups

	Variables	Estimated Mean ± SE			η^2^*-*value	*p*-value^*^
		Baseline	1 week later	2 week later		
O'Leary Index	Saline group	54.54 ± 3.86^aA^	71.30 ± 0.28^aB^	68.96 ± 0.79^aB^	0.325	** < 0.001**
	Chlorhexidine group	51.52 ± 4.79^aA^	61.17 ± 4.37^bA^	60.13 ± 4.37^bA^	0.040	0.259
	*Clematis chinensis* Osbeck group	53.70 ± 2.73^aA^	47.17 ± 3.00^cAB^	40.17 ± 2.96^cB^	0.142	**0.006**
	η^2^*-*value	0.005	0.322	0.410		
	*p*-value^*^	0.851	** < 0.001**	** < 0.001**		
Cariogenic activity	Saline group	83.70 ± 1.80^aA^	71.55 ± 1.09^aB^	76.44 ± 3.26^aAB^	0.184	**0.001**
	Chlorhexidine group	79.19 ± 2.02^aA^	79.58 ± 1.52^bA^	74.17 ± 1.70^aA^	0.082	0.060
	*Clematis chinensis* Osbeck group	80.11 ± 2.02^aA^	67.46 ± 2.58^aB^	62.53 ± 1.58^bB^	0.361	** < 0.001**
	η^2^*-*value	0.043	0.253	0.241		
	*p*-value^*^	0.231	** < 0.001**	** < 0.001**		
Satisfaction	Saline group	1.09 ± 0.06^aA^	1.13 ± 0.07^aA^	1.09 ± 0.06^aA^	0.005	0.859
	Chlorhexidine group	1.22 ± 0.09^aA^	1.43 ± 0.11^aA^	1.22 ± 0.09^aA^	0.051	0.178
	*Clematis chinensis* Osbeck group	1.30 ± 0.12^bA^	4.35 ± 0.21^bB^	4.74 ± 0.14^bB^	0.367	** < 0.001**
	η^2^*-*value	0.042	0.822	0.924		
	*p*-value^*^	0.244	** < 0.001**	** < 0.001**		

Post hoc comparisons using Duncan’s test

Time differences within a group are indicated by uppercase letters (A, B, C)

Group differences at the same time point are indicated by lowercase letters (a, b, c)

*SE* standard error

^*^p-values are determined by ANOVA test (p < 0.05). Bolded p*-*values denote statistical significance

Similarly, no baseline differences in cariogenic activity were found among groups. At week 1, the chlorhexidine group showed higher activity than the saline and *C. chinensis* Osbeck groups (η^2^ = 0.253, *P* < 0.001). By week 2, the *C. chinensis* Osbeck group showed the lowest cariogenic activity, with no significant difference between the saline and chlorhexidine groups (η^2^ = 0.241, *P* < 0.001).

Over time, the saline group exhibited a transient decrease at week 1, followed by an increase at week 2 (η^2^ = 0.184, *P* = 0.001). The chlorhexidine group showed no significant temporal change, whereas the *C. chinensis* Osbeck group demonstrated a significant and sustained decrease from week 1 (η^2^ = 0.361, *P* < 0.001).

Regarding satisfaction with mouthwash use, only the *C. chinensis* Osbeck group showed a significant improvement over time, with mean satisfaction scores exceeding 4 points at both 1 and 2 weeks (η^2^ = 0.367, *P* < 0.001).

### Caries risk levels based on saliva test parameters

Comparative results of salivary tests for the saline, chlorhexidine, and *C. chinensis* Osbeck gargle groups are presented in Table 3. Regarding dental caries-related bacteria, a significant intergroup difference was observed at 2 weeks, with the chlorhexidine group and *C. chinensis* Osbeck extract gargle groups showing lower bacterial levels than the saline group (η^2^ = 0.178, *P* = 0.002). Across time points, significant changes were noted in the saline and *C. chinensis* Osbeck extract groups. In the saline group, bacterial counts decreased at 1 week but increased again at 2 weeks compared to the baseline (η^2^ = 0.360, *P* < 0.001). Conversely, the *C. chinensis* Osbeck extract group showed a continuous decline from 1 to 2 weeks (η^2^ = 0.188, *P* < 0.001).

**Table 3:** Salivary test results

	Variables	Estimated Mean ± SE			η^2^*-*value	*p*-value^*^
		Baseline	1 week later	2 week later		
Dental caries-related bacteria	Saline group	39.57 ± 2.81^aA^	21.17 ± 3.42^aB^	42.96 ± 1.63^aA^	0.360	** < 0.001**
	Chlorhexidine group	31.39 ± 4.20^aA^	28.43 ± 3.41^aA^	27.91 ± 5.40^bA^	0.005	0.835
	*Clematis chinensis* Osbeck group	43.78 ± 4.96^aA^	24.48 ± 3.75^aB^	21.83 ± 4.22^bB^	0.188	** < 0.001**
	η^2^*-*value	0.067	0.031	0.178		
	*p*-value^*^	0.101	0.352	**0.002**		
Acid production capacity	Saline group	71.39 ± 3.11^aA^	77.96 ± 2.74^aA^	75.74 ± 2.72^aA^	0.040	0.263
	Chlorhexidine group	65.26 ± 4.13^aA^	72.48 ± 6.63^aA^	69.48 ± 5.46^aA^	0.013	0.649
	*Clematis chinensis* Osbeck group	69.35 ± 3.04^aA^	49.91 ± 3.74^bB^	48.65 ± 3.25^bB^	0.266	** < 0.001**
	η^2^*-*value	0.024	0.235	0.277		
	*p*-value^*^	0.448	** < 0.001**	** < 0.001**		
Buffering capacity (reverse coding)	Saline group	29.91 ± 1.48^aA^	28.35 ± 2.35^aA^	21.48 ± 1.42^aB^	0.158	**0.003**
	Chlorhexidine group	28.70 ± 1.65^aA^	22.87 ± 3.11^aA^	12.74 ± 3.34^bB^	0.201	** < 0.001**
	*Clematis chinensis* Osbeck group	26.91 ± 3.77^aA^	13.44 ± 2.33^bB^	9.87 ± 2.52^bB^	0.220	** < 0.001**
	η^2^*-*value	0.011	0.200	0.146		
	*p*-value^*^	0.701	** < 0.001**	**0.006**		
Total saliva test score	Saline group	140.00 ± 2.71^aA^	123.26 ± 4.48^aB^	139.30 ± 2.65^aA^	0.192	** < 0.001**
	Chlorhexidine group	125.35 ± 6.01^aA^	123.78 ± 4.67^aA^	110.13 ± 5.13^bA^	0.070	0.090
	*Clematis chinensis* Osbeck group	140.04 ± 8.03^aA^	87.83 ± 3.78^bB^	80.35 ± 6.57^cB^	0.447	** < 0.001**
	η^2^*-*value	0.057	0.420	0.518		
	*p*-value^*^	0.144	** < 0.001**	** < 0.001**		

Post hoc comparisons using Duncan’s test

Time differences within a group are indicated by uppercase letters (A, B, C)

Group differences at the same time point are indicated by lowercase letters (a, b, c)

*SE* standard error

^*^p-values are determined by ANOVA test (p < 0.05). Bolded *p*-values denote statistical significance

For acid production capacity, only the *C. chinensis* Osbeck extract group demonstrated a significant reduction, evident at both 1 week (η^2^ = 0.235, P < 0.001) and 2 weeks (η^2^ = 0.277, *P* < 0.001). This group also showed a progressive decrease over time (η^2^ = 0.266, *P* < 0.001).

As buffering capacity is reverse coded (higher values indicating lower buffering ability), a significant decrease was noted at 1 week in the *C. chinensis* Osbeck extract group (η^2^ = 0.200, *P* < 0.001), and at 2 weeks in both the chlorhexidine group and *C. chinensis* Osbeck extract groups (η^2^ = 0.146, *P* = 0.006). The *C. chinensis* Osbeck extract group exhibited the lowest values, reflecting better buffering capacity, though the difference from the chlorhexidine group was insignificant. Across all groups, buffering capacity improved over time, with the *C. chinensis* Osbeck extract group showing significant improvement by week 1 and sustaining it through week 2 (η^2^ = 0.220, *P* < 0.001).

Analysis of the total score, incorporating all three salivary parameters, revealed no baseline differences among the groups. However, at 1 week, a significant reduction was observed only in the *C. chinensis* Osbeck extract group (η^2^ = 0.420, *P* < 0.001). By 2 weeks, the chlorhexidine group also showed a reduction, but the *C. chinensis* Osbeck extract group demonstrated a more significant decrease (η^2^ = 0.518, *P* < 0.001). Regarding changes over time, the saline group showed a reduction at 1 week but an increase by 2 weeks (η^2^ = 0.192, *P* < 0.001). However, the *C. chinensis* Osbeck extract group exhibited a continuous decline from 1 to 2 weeks (η^2^ = 0.447, *P* < 0.001).

### Quantitative analysis of cariogenic bacteria

Table 4 presents the bacterial counts among the three groups. At baseline, there were no significant differences in *S. mutans* and the *GS* group (comprising *S. mitis, S. sobrinus,* and *Lactobacillus casei*). By week 1, the *C. chinensis* Osbeck extract group showed a significantly greater reduction in both *S. mutans* (η^2^ = 0.102, *P* = 0.028) and *GS* bacteria (η^2^ = 0.146, *P* = 0.006) compared to the chlorhexidine group.

**Table 4:** Difference in caries-causing oral bacteria following gargle application

	Variables	Estimated mean ± SE			η^2^*-*value	*p*-value^*^
		Baseline	1 week later	2 week later		
*Streptococcus mutans*	Saline group	770.57 ± 275.40^aA^	805.44 ± 288.96^aA^	740.91 ± 287.76^aA^	0.000	0.987
	Chlorhexidine group	614.09 ± 288.20^aA^	446.65 ± 209.83^abA^	328.74 ± 165.23^abA^	0.012	0.672
	*Clematis chinensis* Osbeck group	817.17 ± 251.53^aA^	7.39 ± 2.70^bB^	0.09 ± 0.06^bB^	0.241	** < 0.001**
	η^2^*-*value	0.005	0.102	0.102		
	*p*-value^*^	0.859	**0.028**	**0.029**		
*GS* (*Streptococcus mitis, Streptococcus sobrinus,* and *Lactobacillus casei*)	Saline group	1,058,204.78 ± 251,941.50^aA^	1,104,599.22 ± 269,564.99^aA^	1,152,523.74 ± 268,010.23^aA^	0.001	0.968
	Chlorhexidine group	898,392.83 ± 93,279.26^aA^	736,057.22 ± 114,869.67^abA^	732,875.39 ± 101,206.64^aA^	0.025	0.438
	*Clematis chinensis* Osbeck group	836,222.83 ± 157,900.32^aA^	283,744.65 ± 64,383.55^bB^	29,788.22 ± 7760.51^bB^	0.347	** < 0.001**
	η^2^*-*value	0.012	0.146	0.263		
	*p*-value^*^	0.669	**0.006**	** < 0.001**		

Post hoc comparisons using Duncan’s test

Time differences within a group are indicated by uppercase letters (A, B, C)

Group differences at the same time point are indicated by lowercase letters (a, b, c)

^*^p-values are determined by ANOVA test (p < 0.05). Bolded *p*-values denote statistical significance

At 2 weeks, *S. mutans* levels remained similar in the chlorhexidine group but further decreased in the *C. chinensis* Osbeck extract group (η^2^ = 0.102, *P* = 0.029). For the *GS* group, only the *C. chinensis* Osbeck extract group exhibited a significant antibacterial effect (η^2^ = 0.263, *P* < 0.001). Notably, the *C. chinensis* Osbeck extract group demonstrated sustained bacterial reduction from week 1 onward for both *S. mutans* (η^2^ = 0.241, *P* < 0.001) and the *GS* group (η^2^ = 0.347, *P* < 0.001).

## Discussion

Dental caries is a chronic infectious disease caused by acidogenic bacteria metabolizing sugars to produce acids, demineralizing enamel and dentin [19]. Left untreated, it can negatively impact systemic health by impairing masticatory function, causing pain, limiting nutritional intake, and affecting speech. In children and adolescents, it may further hinder growth and development [20]. Moreover, dental caries poses a significant public health burden due to its high treatment costs and recurrence rates [21].

Extensive global research is focused on preventing dental caries. Notably, mouthwashes containing antibacterial agents have demonstrated efficacy in suppressing *S. mutans* and other acid-producing bacteria in dental plaque, thereby reducing caries risk and promoting a healthier oral flora [19]. Regular use of such mouthwashes increases salivary antibacterial activity and helps prevent acidification of the oral environment, contributing to caries prevention [5]. However, long-term use of chemical mouthwashes may lead to adverse gastrointestinal effects such as dry mouth, taste disturbances, mucosal irritation, diarrhea, and vomiting. Commonly reported local side effects include tooth staining, oral papillary discoloration, and mucosal desquamation [22, 23]. Chlorhexidine, a widely used antibacterial ingredient, exhibits broad-spectrum antibacterial activity. However, it can disrupt the balance of the normal oral microbiota [24]. This disruption can alter the microbial ecosystem, promote the selective proliferation of pathogenic bacteria, and potentially increase the risk of antimicrobial resistance [25]. Conversely, naturally derived bioactive compounds are gaining attention for their favorable biocompatibility, fewer side effects, and multifunctional properties, including antibacterial, anti-inflammatory, and antioxidant effects [26]. This study evaluated the antibacterial effects of *C. chinensis* Osbeck extract against primary cariogenic bacteria and compared its efficacy to that of chlorhexidine. Our findings support the potential application of *C. chinensis* Osbeck extract as a natural antibacterial agent in functional mouthwashes for dental caries prevention.

The demographic characteristics of the study participants showed no significant differences across groups, ensuring baseline homogeneity. This study evaluated the effects of a natural *C. chinensis* Osbeck extract-containing mouthwash on dental caries-related indices. Notably, only the *C. chinensis* Osbeck extract group demonstrated a significant reduction in the O’Leary index scores after 1 week, with this improvement persisting through 2 weeks. These results suggest that the extract exhibits antibacterial activity that inhibits plaque formation and disrupts the adhesion of oral pathogens. This observation aligns with previous reports that eugenol, a plant-based antibacterial compound, reduces the pathogenicity of *S. mutans* by inhibiting plaque formation [27]. Furthermore, unlike the chlorhexidine group, which showed no significant change in caries activity, the *C. chinensis* Osbeck extract group exhibited a continuous decline from the first week. This finding likely reflects the suppression of acid production by cariogenic bacteria, consistent with studies using mouthwashes containing *Sambucus williamsii* extract; this ingredient similarly reduced acid production and lowered the risk of caries activity [28]. However, the transient increases in the O’Leary index and cariogenic activity observed in the chlorhexidine group should be interpreted with caution. While meta-analyses provide sufficient evidence supporting chlorhexidine in reducing gingival inflammation among patients with mild gingivitis, they indicate insufficient evidence for its efficacy in individuals with moderate to severe gingival inflammation [29]. Therefore, these fluctuations are more likely attributable to baseline disease severity, the short 2-week study duration, and the limited sample size, rather than to any inherent superiority of the natural product. Accordingly, further well-designed, longer-term studies are warranted to clarify these effects.

Regarding mouthwash satisfaction, only the *C. chinensis* Osbeck extract group showed a gradual increase over time. This finding suggests that natural product-based formulations offer greater user acceptance regarding aesthetics and sensory experience—an important finding given the side effects of conventional chlorhexidine, such as discoloration, halitosis, and mucosal irritation [10]. Compared to chemical mouthwashes with intense flavors, the *C. chinensis* Osbeck extract likely improved user satisfaction by providing antibacterial effects with minimal discomfort related to usability, taste, scent, and oral irritation. A recent study similarly emphasized that user satisfaction is influenced not only by antibacterial efficacy but also by sensory attributes (flavor, aroma, and mouthfeel), safety perceptions, and perceived oral health improvements [30], consistent with the high satisfaction observed in the *C. chinensis* Osbeck group.

Saliva plays a crucial role in oral health by neutralizing acids through its buffering capacity, remineralizing enamel, cleansing food debris, and inhibiting bacterial growth via its enzymatic components. [31] Salivary buffering capacity and acid inhibition are closely related to caries risk. Therefore, this study aimed to evaluate changes in the salivary caries-related indicators to assess how the extract influenced the oral environment.

A significant reduction was observed in the *C. chinensis* Osbeck extract group at 2 weeks for cariogenic bacteria levels. Notably, while the saline group showed a transient decrease followed by a rebound, the *C. chinensis* Osbeck extract group maintained a sustained reduction. This observation suggests that the extract suppresses cariogenic bacterial growth and helps stabilize the salivary microbial balance. Moreover, only the *C. chinensis* Osbeck group exhibited a significant reduction in acid production capacity at 1 and 2 weeks, with differences from other groups emerging as early as week 1. Salivary buffering capacity is a critical defense mechanism that protects teeth from acid and helps prevent dental caries. Although all groups showed significant changes over time, the *C. chinensis* Osbeck extract group demonstrated improved buffering capacity from week 1, which was maintained through week 2. This observation suggests natural product-based mouthwashes stimulate salivary secretion or activate buffering components [32, 33]. These results indicate that *C. chinensis* Osbeck extract may help prevent caries by enhancing the oral acid recovery, inhibiting cariogenic bacterial growth, and consistently improving caries-related indices in saliva. Overall, it appears to have potential as a natural ingredient with beneficial effects related to dental caries. Dental caries is a polymicrobial infection involving Gram-positive bacteria, including *S. mutans, S. mitis, S. sobrinus*, and *L. casei* [34]. *S. mutans* and *S. mitis* contributes to early plaque formation, providing a foundation for the colonization of other bacteria and the development of a cariogenic environment [35]. *S. sobrinus* produces acid more rapidly and attaches more readily than *S. mutans*, accelerating caries progression [36]. Furthermore, *L. casei* is commonly observed in deep carious lesions or areas extending to the pulp, playing a key role in advanced-stage caries [37]. These species, collectively classified within the Gram-positive *GS* group, are known to amplify pathogenicity through their synergistic interactions within dental plaque. This study demonstrated that a mouthwash containing *C. chinensis* Osbeck extract effectively reduced the levels of *S. mutans,* the primary cariogenic bacterium, and *S. mitis, S. sobrinus*, and *L. casei* in the *GS* group. A significant antibacterial effect was observed at 1 week and persisted through 2 weeks. Compared to the chemical agent chlorhexidine, *C. chinensis* Osbeck extract showed a stronger and more sustained anticariogenic effect, particularly over time, indicating selective action against both single bacterial species and broader cariogenic bacterial groups.

These findings suggest that *C. chinensis* Osbeck extract may improve clinical indicators related to dental caries while demonstrating acceptable effectiveness and safety as a natural mouthwash. Throughout the study period, both the *C. chinensis* Osbeck extract and chlorhexidine groups were well tolerated, and no notable adverse effects, such as changes in oral mucosa, taste disturbances, or gastrointestinal symptoms, were observed. However, the relatively short 2-week observation period limits the assessment of long-term effects. Future studies should include extended follow-up and evaluate efficacy in specific populations such as children and adolescents, older adults, and patients undergoing orthodontic treatment. Furthermore, while chlorhexidine was used as a chemical comparator, its effects were not assessed against commercially available natural antibacterial agents, representing another limitation. Additionally, this study primarily focused on the antibacterial effects of *C. chinensis* Osbeck extract against major cariogenic bacteria, and the impact on the broader oral microbiota, particularly in healthy individuals without dental problems, was not assessed. Therefore, the potential for dysbiosis or disruption of overall oral microbial homeostasis cannot be fully excluded, and future studies should investigate these effects to ensure the safety and ecological balance of the oral microbiome. Given that this was a clinical intervention study, future trials should adopt larger sample sizes and quantitative assessments of salivary flow and pH to strengthen causal inference and enhance generalizability. Despite these limitations, the present findings suggest that the *C. chinensis* Osbeck extract has potential as a natural mouthwash component that may contribute to the improvement of the oral environment. Its antibacterial and caries-associated properties indicate possible utility in preventive oral care, although further clinical validation is warranted before broader application. The *C. chinensis* Osbeck extract group showed significant improvements in primary dental caries indicators, such as O’Leary index, cariogenic activity, salivary caries-related bacteria, acid production, and buffering capacity, as well as reductions in major cariogenic bacteria after 1 week of use, with these effects persisting through 2 weeks. A mouthwash containing *C. chinensis* Osbeck extract could potentially support oral health management as a safe and effective adjunct, complementing existing chemical mouthwashes and contributing to the prevention of dental caries.

## Supplementary Information

Additional file 1

Additional file 2

## Data Availability

The datasets generated during and/or analysed during the current study are available from the corresponding author on reasonable request.

## Abbreviations

*C. chinensis* Osbeck: *Clematis chinensis* Osbeck
*S. **mutans*: *Streptococcus* *mutans*
*L **casei*: *Lactobacillus* *casei*
GTFs: Glycosyltransferases
CPC: Cetylpyridinium chloride
*S. Williamsii*: *Sambucus williamsii* Var. *coreana* Nakai
