# Supplementary figures and images for: Clinical evaluation of a Clematis chinensis Osbeck–containing mouthwash for the prevention of dental caries: a randomized, controlled clinical trial
Source: Chin Med. 2025 Nov 24;20:199. doi: 10.1186/s13020-025-01258-z (PMC12642278; doi:10.1186/s13020-025-01258-z)

O'Leary Index

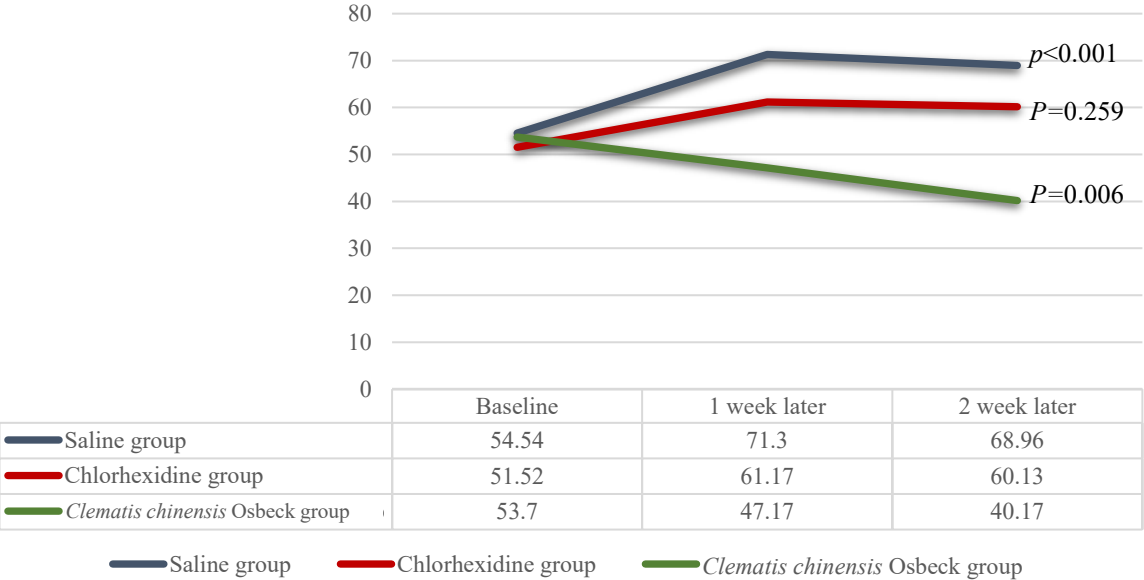

Cariogenic activity

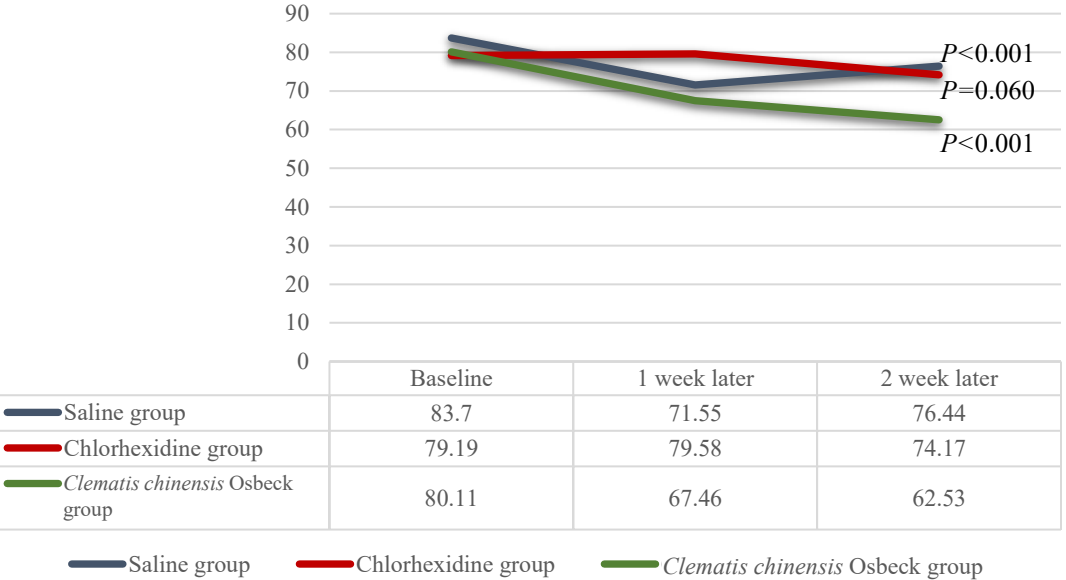

Satisfaction

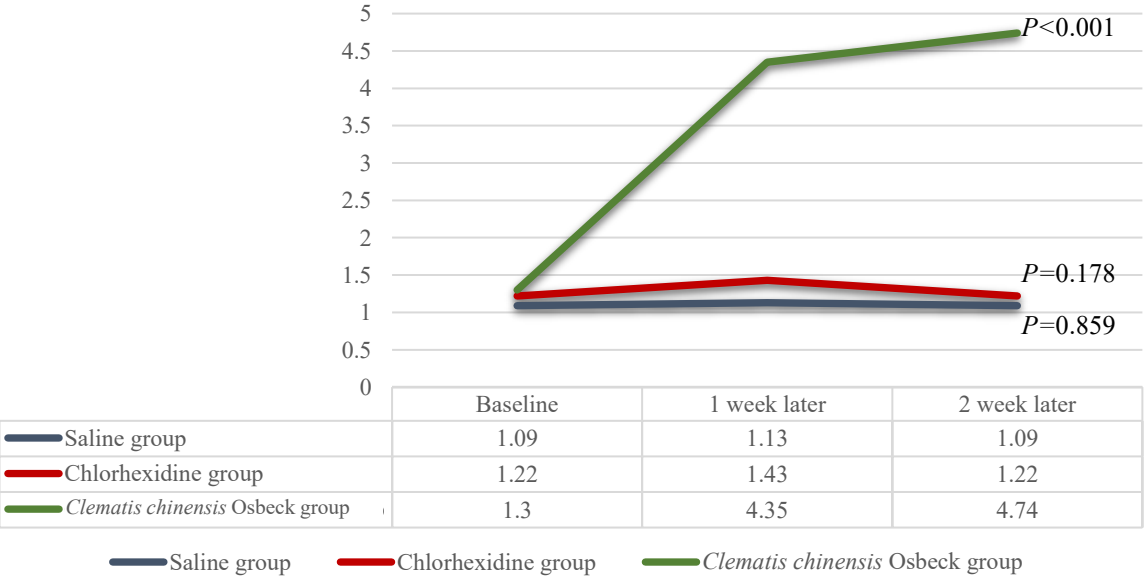

Supplement: Supplementary file 1 — Additional file 1 [file 13020_2025_1258_MOESM1_ESM.pdf]
