# Supplementary material for: Clinical evaluation of a Clematis chinensis Osbeck–containing mouthwash for the prevention of dental caries: a randomized, controlled clinical trial
Source: Chin Med. 2025 Nov 24;20:199. doi: 10.1186/s13020-025-01258-z (PMC12642278; doi:10.1186/s13020-025-01258-z)
Supplement: Supplementary file 2 — Additional file 2 [file 13020_2025_1258_MOESM2_ESM.docx]

**Supplementary table 1: Primers and probes used in the real-time polymerase chain reaction assays.**

| Bacteria | Target genes | Primers/Probe sets | Amplicon size (bp) |
| --- | --- | --- | --- |
| *Streptococcus mutans* | mannitol-specific enzyme II (mtlA) gene | 5′-CAGCGCATTCAACACAAGCA-3′ 103  5′-TGTCCCATCGTTGCTGAACC-3′  5′-HEX-TGCGGTCGTTTTTGCTCATGG-BHQ1–3′ | 103 |
| *GS* group  (*Streptococcus mitis*, *Streptococcus sobrinus*, *Lactobacillus casei*) | 16S  ribosomal RNA gene | GTACAACGAGTCGCAAGCCG  TACAAGGCCCGGGAACGTAT  [5FAM]TAATCGCGGATCAGCACGCC[3BHQ1] | 149 |

bp: base pairs
